# *Carica papaya* L. Hexane Fraction Induces Apoptotic Cell Death in Colorectal Cancer Cells via PI3K/AKT Suppression and Impaired Late-Stage Autophagic Flux

**DOI:** 10.3390/nu18213557

**Published:** 2026-10-28

**Authors:** Rambukkana Maggonage Thiruni Dananjana Perera, Su-Lim Kim, Ji-Hyang Kim, Kyeoung Cheol Kim, Kang-Ui Tak, Seung-Min Lee, Chankyu Lim, Dong-Sun Lee

**Affiliations:** 1 Interdisciplinary Graduate Program in Advanced Convergence Technology & Science, Jeju National University, Jeju 63243, Republic of Korea; thiruniperera@stu.jejunu.ac.kr (R.M.T.D.P.); ai202619304@stu.jejunu.ac.kr (K.-U.T.); ai202619302@stu.jejunu.ac.kr (S.-M.L.); 2 Graduate Program for Bio-Health/Innovative Drug Development Using Subtropical Bio-Resources, Jeju National University, Jeju 63243, Republic of Korea; ksl1101@jejunu.ac.kr; 3 Bio-Health Materials Core-Facility Center, Jeju National University, Jeju 63243, Republic of Korea; seogwi12@jejunu.ac.kr (J.-H.K.); cheolst2@jejunu.ac.kr (K.C.K.); 4 Faculty of Biotechnology, College of Applied Life Sciences, Jeju National University, Jeju 63243, Republic of Korea; 5 Research Institute of Climate Change and Agriculture, National Institute of Horticultural and Herbal Science, Rural Development Administration, Jeju 63243, Republic of Korea; badang05@korea.kr

**Keywords:** *Carica papaya* L., colorectal cancer, PI3K/AKT signaling pathway, autophagic flux blockade, reactive oxygen species

## Abstract

**Background/Objectives:** Colorectal cancer (CRC) remains a major contributor to cancer-associated deaths globally. *Carica papaya* L., a tropical fruit species, is reported to contain a wide range of biological properties, along with potential anticancer effects. However, studies evaluating the therapeutic potential and molecular mechanisms of Korean *Carica papaya* L. cultivars against CRC are limited. Therefore, this study investigated the effect of the hexane fraction of *Carica papaya* L. on the induction of apoptosis and the underlying mechanisms associated with this effect in human CRC cells. **Methods:** We performed several in vitro experiments, including cell viability, apoptosis, assessment of autophagic vacuoles, Western blotting, and analysis of autophagic flux. Subsequently, we verified the findings in an animal model of CRC induced by azoxymethane/dextran sulfate sodium. **Results:** BR 56 Hex F reduced cell viability and induced caspase-7/9-dependent apoptosis accompanied by intracellular and mitochondrial reactive oxygen species elevation. Mechanistically, BR 56 Hex F suppressed phosphatidylinositol 3-kinase (PI3K; p85/p110), p-AKT (Ser473/Thr308), and mammalian target of rapamycin signaling. Although autophagosome formation was initiated, the late-stage autophagic flux was impaired, as evidenced by p62 accumulation, LAMP-1 depletion, and autophagosome accumulation. In vivo, oral administration of BR 56 *C. papaya* L. extract significantly attenuated azoxymethane/dextran sulfate sodium-induced colon shortening, splenomegaly, histopathological damage, and colonic PI3K and p-AKT expression. **Conclusions:** BR 56 Hex F inhibited CRC growth by suppressing PI3K/AKT signaling and impairing late-stage autophagic flux, representing a potential natural candidate for CRC treatment.

## 1. Introduction

Colorectal cancer (CRC) is considered one of the most substantial global health challenges. Although surgery, chemotherapy, targeted agents, and immunotherapy are used in clinical management, their effectiveness can be restricted by adverse effects [1]. CRC progresses through four main clinical stages as localized lesions advance into metastatic disease, where tumor cells disseminate through the circulation to distant organs throughout the body [2]. Current treatment strategies include surgical resection, chemotherapy, targeted therapy, and immunotherapy [3]. However, the clinical effectiveness of these approaches can be limited by treatment related side effects, tumor heterogeneity, and the development of resistance. These limitations highlight the need for novel therapeutic agents with novel mechanisms of action and improved efficacy [4]. Consequently, identification of novel anticancer agents, particularly those derived from natural products, has attracted considerable interest. Natural products are a valuable reservoir of biologically active substances with a broad range of pharmacological effects and hold promise for developing novel approaches to address the molecular complexity of colorectal cancer (CRC) [5].

*Carica papaya* L. is a tropical fruit crop in the family *Caricaceae,* originally native to tropical America and now cultivated worldwide, including Asia and Africa. This fruit is valued for its nutritional, medicinal, and health benefits. Environmental factors such as temperature, sunlight, rainfall, and soil conditions strongly influence papaya growth and phytochemical composition. Optimal climates (21–33 °C), adequate sunlight, and nutrient-rich soils support the accumulation of beneficial compounds, whereas temperature stress and water deficiency may reduce yield and degrade sensitive nutrients. Although papaya cultivation has expanded to non-tropical regions, such as Japan and Korea, through greenhouse production, the effects of controlled cultivation conditions on fruit quality and phytochemical properties have not been sufficiently evaluated [6]. The papaya fruit is recognized for its diverse array of bioactive constituents, which have been shown to confer various pharmacological benefits, including antioxidant, anti-inflammatory, anticancer, antibacterial, anti-aging, antidiabetic, anti-obesity, and wound-healing activities [6]. Additionally, several studies have reported the anticancer potential of papaya extracts; however, the underlying molecular mechanisms, particularly regarding autophagy regulation and the oncogenic signaling pathways involved in colorectal cancer progression, remain comparatively underexplored.

Among the signaling pathways implicated in colorectal tumorigenesis, the phosphatidylinositol 3-kinase/protein kinase B (PI3K/AKT) signaling cascade is commonly dysregulated in colorectal tumor development, contributing to uncontrolled proliferation, resistance to apoptosis, metabolic reprogramming, and resistance to chemotherapy [7]. Phosphatidylinositol 3-kinase (PI3K) is an intracellular phosphatidylinositol kinase that is activated by cell surface receptors to produce a secondary messenger, which leads to the activation of downstream protein kinase B (AKT). AKT phosphorylation further promotes the activation of rapamycin (mTOR) and triggers tumor proliferation and progression. Consequently, the PI3K/AKT/mTOR pathway has been documented as a major antitumor target [7]. The PI3K/AKT/mTOR signaling cascade is an important regulator of autophagy, linking pathways that support tumor cell survival with mechanisms involved in adaptation to cellular stress [8].

Autophagy is an intracellular degradation pathway that maintains cellular homeostasis by delivering damaged proteins and organelles to the lysosomes for degradation and recycling. This plays a complex role in the biology of cancer, as its dysregulation, specifically by blocking late-stage autophagic flux, has emerged as an effective therapeutic strategy for triggering cancer cell death [9,10]. Interruption of autophagosome–lysosome fusion or impairment of lysosomal degradation leads to the toxic accumulation of cellular debris, damaged mitochondria, autophagosomes, ubiquitinated protein aggregates, and the autophagy receptor p62/SQSTM1, thereby promoting cellular stress and, in some contexts, apoptotic cell death [11]. Thus, the interplay between PI3K/AKT signaling and autophagy regulation is particularly relevant to cancer therapy. The inhibition of PI3K/AKT/mTOR signaling can stimulate autophagic responses as a cellular adaptation mechanism, while the impairment of late-stage autophagic degradation can prevent the completion of the autophagic process, thereby converting this protective response into a cytotoxic mechanism [12]. Consequently, compounds capable of simultaneously suppressing PI3K/AKT signaling and disrupting autophagic flux may provide an effective strategy for inducing colorectal cancer cell death [13,14].

Despite extensive studies on the biological activities of papaya, the anticancer potential of solvent fractions obtained from domestically cultivated papaya varieties in Korea remains unknown. Specifically, whether Korean-grown *C. papaya* L. cultivars exert anticancer effects in CRC by regulating the PI3K/AKT signaling axis and autophagic flux remains unclear. Therefore, the current study investigated the anticancer activity of the hexane fraction (BR 56 Hex F) prepared from the immature (green) *C. papaya* L. cultivar BR 56, which is cultivated in Korea. We systematically evaluated the antiproliferative and proapoptotic effects of BR 56 Hex F on human CRC cells (HT-29 and HCT-116), focusing specifically on its capacity to inhibit PI3K/AKT signaling and induce late-stage autophagic flux blockade.

Preliminary cell viability screening comparing mature (BR 56 Yellow) and immature (BR 56 Green) fruit fractions revealed that the BR 56 Green hexane fraction exhibited the highest cytotoxic activity against HT-29 cells among the fractions tested (Figures S1 and S2). Consequently, the immature fruit (BR 56 Green) hexane fraction was selected for all subsequent in vitro experiments.

## 2. Materials and Methods

### 2.1. Materials

Immature *C. papaya* L. fruits harvested between August and October 2024 before reaching full maturity were obtained from the Research Institute of Climate Change and Agriculture, National Institute of Horticultural and Herbal Science, Rural Development Administration, Jeju, Republic of Korea. The fruits were obtained from an experimental papaya breeding line provisionally designated BR 56 during the cultivar development process. The BR breeding line was derived from an artificial cross between Bolivia papaya and Redlady, with the numerical suffix indicating the individual tree identifier. The immature fruits were designated as BR 56 Green and were collected before reaching full maturity. For permanent traceability and reference, representative dried plant specimens and retained extract samples were catalogued and deposited in the specimen repository of the Biopharmaceutical Materials Research Laboratory, Jeju National University, under the voucher/reference number JNU-2024-BR 56(G).

### 2.2. Preparation and Fractionation of the Carica papaya L. Fruit Extract

Fresh *Carica papaya* L. fruits were freeze-dried and pulverized into a fine powder. A 100 g portion of the powdered material was extracted with 2 L of 70% (*v*/*v*) ethanol under continuous agitation for 24 h. The resulting suspension was passed through ADVANTEC No. 2 filter paper (Toyo Roshi Kaisha, Ltd. (ADVANTEC), Tokyo, Japan), and the collected filtrate was concentrated using a rotary evaporator maintained at 50 °C. The concentrated extract was then redispersed in 100 mL of distilled water and subjected to sequential liquid–liquid partitioning with 100 mL portions of *n*-hexane, ethyl acetate, and *n*-butanol, in ascending order of solvent polarity. Each organic fraction was concentrated by rotary evaporation at 50 °C and subsequently dried under a nitrogen stream. This procedure yielded the crude ethanolic extract, *n*-hexane, ethyl acetate, and *n*-butanol fractions, together with the remaining aqueous fraction. Each fraction was then dissolved in DMSO at a concentration of 100 mg/mL for use in future experiments. The *n*-hexane fraction obtained from immature fruits of the BR 56 papaya breeding line is hereafter referred to as BR 56 Hex F.

### 2.3. Drugs

*N*-Acetyl-L-cysteine (NAC; Cat. No. A7250), sodium carboxymethylcellulose (Cat. No. C9481), and 5-fluorouracil (5-FU; Cat. No. F6627) were obtained from Sigma-Aldrich, Inc. (St. Louis, MO, USA). Chloroquine (Cat. No. HY-17589A), Z-VAD-FMK (Cat. No. HY-16658B), and bafilomycin A1 (BafA1) were purchased from MedChemExpress LLC (Monmouth Junction, NJ, USA). Stock solutions of BafA1, Z-VAD-FMK, and chloroquine were prepared in dimethyl sulfoxide (DMSO). NAC was resuspended in distilled water (pH 7.5), whereas 5-FU was dissolved in sterile phosphate-buffered saline (PBS) immediately before administration.

### 2.4. Cell Culture

HT-29 and HCT-116 human colorectal carcinoma cells were sourced from the Korea Cell Line Bank (KCLB, Seoul, Republic of Korea). For routine maintenance, the cells were grown in RPMI medium (Corning, Inc., Corning, NY, USA) containing 10% (*v*/*v*) fetal bovine serum (GenDEPOT, Katy, TX, USA) and 1% (*v*/*v*) penicillin–streptomycin (Gibco, Thermo Fisher Scientific Inc., Waltham, MA, USA). Cell growth and maintenance were carried out at 37 °C in a humidified incubator with a 5% CO_2_ atmosphere.

### 2.5. Cell Viability Assay

Cells were seeded at a density of 1.5 × 10^5^ cells/mL and cultured for 48 h. The cells were treated with BR 56 Hex F for an additional 24 h. After treatment, cell viability was assessed using EZ-CYTOX cell viability reagent (DoGenBio, Seoul, Republic of Korea) according to the manufacturer’s protocol. Then absorbance was recorded at 450 nm using a SpectraMax iD5 microplate reader (Molecular Devices, San Jose, CA, USA) located at the Bio-Health Materials Core Facility, Jeju National University, Republic of Korea.

### 2.6. Apoptosis Assay

Apoptotic cell populations following treatment with BR 56 Hex F were analyzed by flow cytometry using an APC Annexin V/propidium iodide (PI) apoptosis detection kit (BioLegend, Inc., San Diego, CA, USA), according to the manufacturer’s instructions. For each sample, at least 5000 events were acquired with a BD Accuri C6 flow cytometer (BD Biosciences, Franklin Lakes, NJ, USA) at the Bio-Health Materials Core Facility of Jeju National University. The acquired data were analyzed using BD Accuri C6 software.

### 2.7. Hoechst Staining

Following the specified duration of BR 56 Hex F treatment, cells were incubated with Hoechst 33342 (1 μg/mL; Thermo Fisher Scientific, Waltham, MA, USA) for 15 min at room temperature in the absence of light. Images were subsequently acquired using a Lionheart FX phase-contrast fluorescence microscope (BioTek, Winooski, VT, USA).

### 2.8. Assessment of Autophagic Vacuoles by Cyto-ID Assay

Cells were maintained at a density of 1.5 × 10^5^ cells/mL for 48 h before exposure to BR 56 Hex F. After treatment, autophagy-associated vesicles were labeled using the CYTO-ID^®^ Autophagy Detection Kit (Enzo, NY, USA), with incubation at 37 °C for 30 min under light-protected conditions. Fluorescence signals corresponding to autophagic vacuoles were examined using a Lionheart FX imaging system (BioTek, Winooski, VT, USA).

### 2.9. Western Blotting

Following culture medium removal, the cells were washed twice with ice-cold phosphate-buffered saline (PBS; pH 7.4). Cellular proteins were extracted using radioimmunoprecipitation assay (RIPA) buffer supplemented with sodium fluoride (NaF), sodium orthovanadate (Na_3_VO_4_), and a protease inhibitor cocktail. The lysates were kept on ice for 15 min, then centrifuged at 15,000 rpm for 10 min at 4 °C. We collected the resulting supernatants for protein quantification using a BCA Protein Assay Kit (Biomax Co., Ltd., Gyeonggi-do, Republic of Korea). Protein samples containing equal amounts of total protein were separated by molecular weight using SDS-PAGE and blotted onto PVDF membranes (MilliporeSigma, Burlington, MA, USA). To minimize nonspecific antibody binding, the membranes were incubated with EzBlock Chemi solution ATTO Corporation, Tokyo, Japan) for 30 min at room temperature. The membranes were then exposed to the appropriate primary antibodies overnight at 4 °C. After washing, the membranes were incubated for 1 h with IRDye 680RD goat anti-mouse IgG or IRDye 800CW goat anti-rabbit IgG secondary antibodies (LI-COR, NE, USA). Immunoreactive bands were detected and quantified using an Odyssey CLx imaging system (LI-COR, Lincoln, NE, USA).

The primary antibodies used included antibodies against caspase-7 (9492S), caspase-9 (9502S), caspase-3 (9662S), SQSTM1/p62, LC3A/B (D3U4C), PI3 kinase p85 (19H8), phospho-AKT (Ser473; D9E) XP, phospho-AKT (Thr308; D25E6) XP, and AKT (9272), all purchased from Cell Signaling Technology (CST)(Danvers, MA, USA). The PI3 kinase p110α antibody (A22730) was obtained from ABclonal Technology (Wuhan, China), whereas antibodies against β-actin and LAMP-1 (H4A3) were obtained from Santa Cruz Biotechnology (Dallas, TX, USA).

### 2.10. Detection of Cellular and Mitochondrial ROS

Cells were seeded into 96-well plates at a concentration of 1.5 × 10^5^/mL and maintained for 48 h before exposure to BR 56 Hex F. Following treatment, intracellular reactive oxygen species (ROS) and mitochondrial superoxide were examined using CellROX Green and MitoSOX Red fluorescent probes, respectively. For intracellular ROS detection, the cells were incubated with CellROX Green (Thermo Fisher Scientific, Waltham, MA, USA) at 37 °C for 30 min in the dark. Mitochondrial superoxide was assessed using MitoSOX Red (Thermo Fisher Scientific, Waltham, MA, USA), following the manufacturer’s protocol, with incubation for 30 min at 37 °C under dark conditions. Fluorescence signals were subsequently examined using a Lionheart FX phase-contrast fluorescence microscope (BioTek Instruments, Inc., Winooski, VT, USA).

### 2.11. Analysis of Autophagic Flux

For autophagic flux analysis, cells were transiently transfected with the mRFP-GFP-LC3 reporter plasmid (Addgene, plasmid #21074; Watertown, MA, USA) using Lipofectamine 3000 (Invitrogen, Thermo Fisher Scientific Inc., Waltham, MA, USA). Following 48 h of transfection, the cells underwent designated treatments for a further 24 h and subsequently fixed with 4% paraformaldehyde. LC3-positive puncta and their fluorescence signals were examined using a Lionheart FX imaging system (BioTek Instruments, Inc., Winooski, VT, USA).

### 2.12. Mice

Male ICR mice aged five weeks were purchased from an accredited laboratory animal supplier and allowed to acclimate for seven days before the experiment. The animals were housed under regulated environmental conditions, with the temperature maintained at 22 ± 2 °C and relative humidity at 50–60%. A 12 h light/12 h dark cycle was provided, and standard chow and drinking water were available ad libitum. Animal care and experimental procedures complied with applicable animal welfare guidelines. The study protocol was reviewed and approved by the Jeju National University Animal Ethics Committee (approval no. 2025-0018).

### 2.13. AOM/DSS Animal Model Establishment

After one week of acclimatization, the mice (*n* = 10/group) were randomly allocated to four experimental groups: (1) negative control, (2) azoxymethane (AOM)/dextran sulfate sodium (DSS) control, (3) BR 56 *C. papaya* L. extract, and (4) positive control. Colitis-associated CRC was induced in an AOM/DSS model. At the third week, mice were given a single intraperitoneal injection of azoxymethane (AOM; 10 mg/kg body weight). After a 7-day interval, 2% (*w*/*v*) dextran sulfate sodium (DSS) was provided in the drinking water for 7 days, followed by 14 days of normal drinking water. This DSS administration and recovery schedule was repeated for three cycles to establish chronic colonic inflammation and promote colorectal tumor development. Mice were administered the BR 56 *C. papaya* L. fruit extract orally by gavage (i.g., 400 mg/kg/day) starting on the day of AOM injection (week 3), continued daily until the end of the 14-week experimental period. The negative and AOM/DSS model control groups received an equal volume of 0.5% CMC-Na as a vehicle control. The positive control group received 5-fluorouracil (5-FU) intraperitoneally at 35 mg/kg twice weekly.

Body weight, rectal bleeding, and clinical signs were monitored weekly throughout the study period. Fresh fecal samples were collected weekly for subsequent analyses. At week 14, the mice were euthanized by gradual exposure to carbon dioxide (CO_2_) in accordance with institutional animal care guidelines. Following euthanasia, the colon, cecum, and spleen were carefully excised. Colon length was measured and evaluated for tumor number and size before processing for histopathological and molecular analyses. The cecum and spleen were collected, weighed, and preserved for histological and biochemical analyses.

### 2.14. Statistical Analysis

All in vitro experiments were performed in three independent biological replicates (*n* = 3). All data are presented as the mean ± SD. Data were examined using one-way analysis of variance (ANOVA) with Dunnett’s multiple comparison test or two-way ANOVA with Tukey’s multiple comparison test. *p*-values are represented as follows: * *p*  <  0.05, ** *p*  <  0.01, *** *p*  <  0.001, and **** *p*  <  0.0001, while *p* ≥ 0.05 was not considered significant (ns). All data were analyzed using GraphPad Prism 8 software (CA, USA).

## 3. Results

### 3.1. BR 56 Papaya Hexane Fraction Induces Apoptotic Cell Death in Colon Cancer Cells

**Figure 1:**
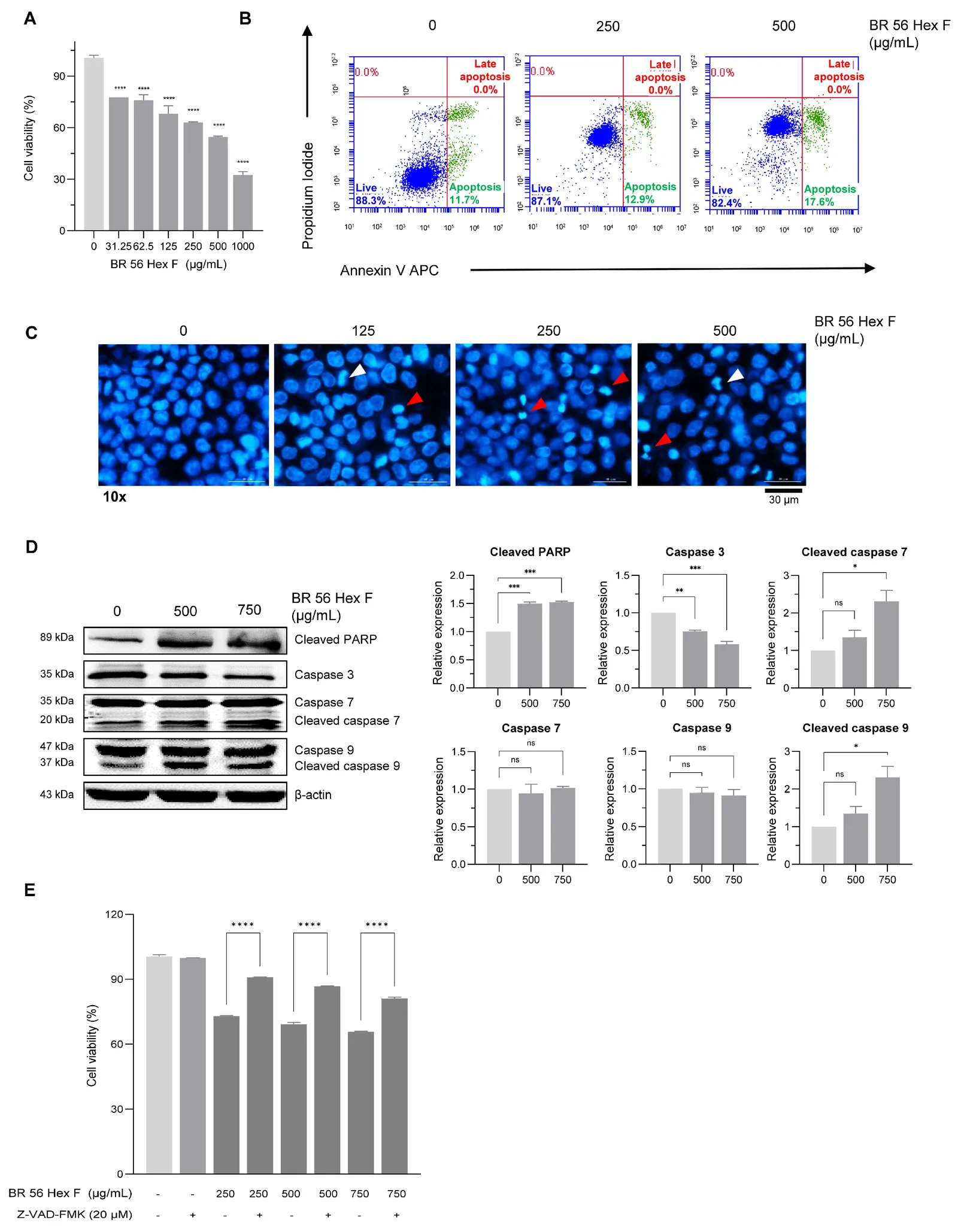
The hexane fraction of BR 56 Green *Carica papaya* L. induces apoptotic cell death in HT-29 colon cancer cells. (**A**) Effects of the BR 56 Hex F on HT-29 cell viability after 24 h of treatment, as determined by the WST assay. (**B**) Representative flow cytometric analysis and quantification of Annexin V/PI staining showing apoptosis in HT-29 cells following treatment with BR 56 Hex F. (**C**) Morphological changes associated with apoptosis were observed by fluorescence microscopy after Hoechst 33342 staining. Arrows indicate apoptotic cells exhibiting nuclear fragmentation (white arrows) and chromatin condensation (red arrows). Magnification × 10; scale bar = 30 μm. (**D**) Protein expression levels of apoptosis-related markers, including caspase-3, caspase-9, cleaved caspase-9, caspase-7, cleaved caspase-7, and PARP, were analyzed by Western blotting after 24 h of treatment. (**E**) Effect of Z-VAD-FMK on BR 56 Hex F-induced cytotoxicity in HT-29 cells. Densitometric analysis was performed using Image Studio software. Data are presented as mean ± SD from three independent biological experiments (*n* = 3). Statistical significance was determined using one-way ANOVA followed by Tukey’s multiple comparisons test. * *p*  <  0.05, ** *p*  <  0.01, *** *p*  <  0.001, and **** *p*  <  0.0001 compared with the control.; ns, not significant.

The viability of HT-29 colon cancer cells treated with various concentrations of the BR 56 hexane fraction was examined using a WST assay. The results showed that HT-29 cells were inhibited in a concentration-dependent manner (Figure 1A). Thus, BR 56 Hex F concentrations of 125, 250, and 500 μg/mL were used in subsequent experiments. Next, we examined BR 56 Hex F-induced apoptosis at different concentrations using Annexin V and propidium iodide (PI) double staining (Figure 1B). Treatment with 250 μg/mL BR 56 Hex F minimally increased the apoptotic cell population to 12.9%, while treatment with 500 μg/mL further increased the apoptotic cell percentage to 17.6%, compared to control cells. These results demonstrated that BR 56 Hex F induces apoptosis in a concentration-dependent manner, indicating that apoptosis is one of the primary mechanisms of BR 56 Hex F-induced cytotoxicity.

Nuclear morphology was evaluated in HT-29 cells using Hoechst 33342 staining after BR 56 Hex F treatment. As shown in Figure 1C, untreated cells maintained intact nuclei with evenly distributed chromatin (NC). In contrast, BR 56 Hex F-treated cells exhibited concentration-dependent increases in chromatin condensation (CC) and nuclear fragmentation (NF). These nuclear abnormalities provide further evidence supporting the induction of apoptosis by BR 56 Hex F in HT-29 cells.

To further investigate the molecular mechanisms underlying BR 56 Hex F-induced apoptosis, Western blot analysis was performed to examine the expression of cleaved PARP, caspase-3, caspase-9, cleaved caspase-9, caspase-7, and cleaved caspase-7, thereby assessing the involvement of caspase-dependent apoptotic signaling. Treatment with BR 56 Hex F markedly increased cleaved PARP expression, particularly at 500 and 750 μg/mL (Figure 1D). Western blot analysis revealed the presence of full-length caspase-9 and caspase-7 across all treatment groups (Figure 1D). In contrast, the abundance of their cleaved forms increased significantly in a concentration-dependent manner following BR 56 Hex F treatment. Meanwhile, full-length caspase-3 protein levels remained stable. These findings suggest that BR 56 Hex F-induced cell death is associated with PARP cleavage and involves the activation of both caspase-9 and caspase-7, whereas the involvement of caspase-3 appears limited.

HT-29 cells were then pretreated with 20 μM Z-VAD-FMK, a pan-caspase inhibitor, for 1 h before exposure to BR 56 Hex F to further confirm the involvement of caspase-dependent apoptosis in the cytotoxic activity of BR 56 Hex F. Z-VAD-FMK was maintained at a final concentration of 20 μM throughout the treatment period. After 24 h of treatment, cell viability was assessed (Figure 1E). Z-VAD-FMK significantly attenuated BR 56 Hex F-induced loss of cell viability at 250, 500, and 750 μg/mL BR 56 Hex F, increasing cell viability from approximately 73%, 70%, and 66% to 91%, 86%, and 80%, respectively. These results demonstrated that the inhibition of caspase activity partially rescued BR 56 Hex F-induced cytotoxicity, supporting the involvement of caspase-dependent apoptosis in the antitumor activity of BR 56 Hex F.

### 3.2. BR 56 Papaya Hexane Fraction Induces Oxidative Stress in Colon Cancer Cells

**Figure 2:**
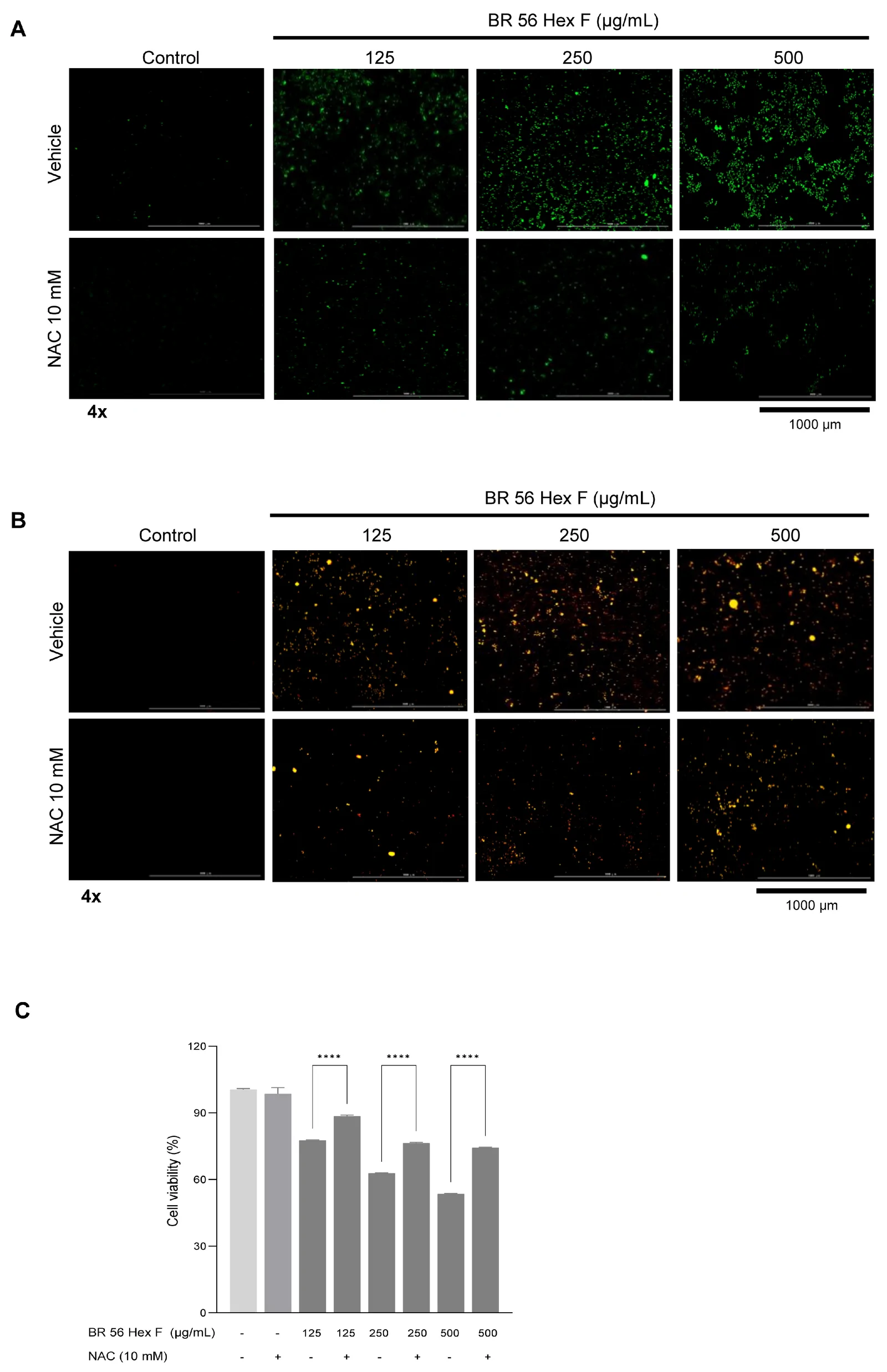
The hexane fraction of BR 56 Green *Carica papaya* L. induces oxidative stress in HT-29 colon cancer cells. (**A**) Intracellular ROS levels were detected using CellROX™ Green staining after 24 h of treatment with the hexane fraction of BR 56 Hex F, with or without 10 mM NAC pretreatment. Images were captured by fluorescence microscopy at ×4 magnification; scale bar = 1000 µm. (**B**) Mitochondrial superoxide production was evaluated using MitoSOX™ Red staining following 24 h of treatment with BR 56 Hex F and visualized by fluorescence microscopy. Images were obtained at ×4 magnification; scale bar = 1000 µm. (**C**) Protective effect of 10 mM NAC against BR 56 Hex F-induced cytotoxicity in colon cancer cells, as shown by a cell viability assay. Data are presented as mean ± SD from three independent biological experiments (*n* = 3). Statistical significance was determined using one-way ANOVA followed by Tukey’s multiple comparisons test. **** *p* <  0.0001 compared with the control.

Intracellular ROS levels were evaluated using CellROX fluorescence staining. Figure 2A shows a concentration-dependent increase in CellROX Green fluorescence following BR 56 Hex F exposure, suggesting enhanced intracellular reactive oxygen species (ROS) accumulation. A detectable increase was observed at 125 μg/mL, with increasing fluorescence at 250 and 500 μg/mL, suggesting progressively increased oxidative stress. To examine the involvement of ROS in this response, cells were pretreated with 10 mM NAC for 2 h before BR 56 Hex F exposure. NAC pretreatment substantially attenuated the fluorescence intensity compared with BR 56 Hex F treatment alone, supporting the involvement of ROS in BR 56 Hex F-induced oxidative stress. The increase in CellROX fluorescence following BR 56 Hex F exposure, together with its attenuation by NAC pretreatment, suggests that redox imbalance contributes to the cellular response observed in HT-29 cells. Subsequently, MitoSOX Red staining was performed to observe mitochondrial superoxide generation after BR 56 Hex F treatment in HT-29 cells. MitoSOX fluorescence increased significantly with increasing BR 56 Hex F concentrations compared with the untreated control, indicating enhanced mitochondrial superoxide accumulation (Figure 2B). To further confirm the involvement of ROS in BR 56 Hex F-induced cytotoxicity, cells were pretreated with the ROS scavenger (NAC, 10 mM) for 2 h prior to BR 56 Hex F exposure. NAC significantly increased cell viability compared with BR 56 Hex F treatment alone (Figure 2C), indicating partial recovery from BR 56 Hex F-induced cytotoxicity. These findings indicate that BR 56 Hex F caused ROS production and that scavenging ROS with NAC attenuates BR 56 Hex F-induced cytotoxicity, supporting the potential involvement of oxidative stress in the observed cytotoxicity.

### 3.3. BR 56 Papaya Hexane Fraction Induced a Blocked Autophagic Flux via Lysosomal Degradation

**Figure 3:**
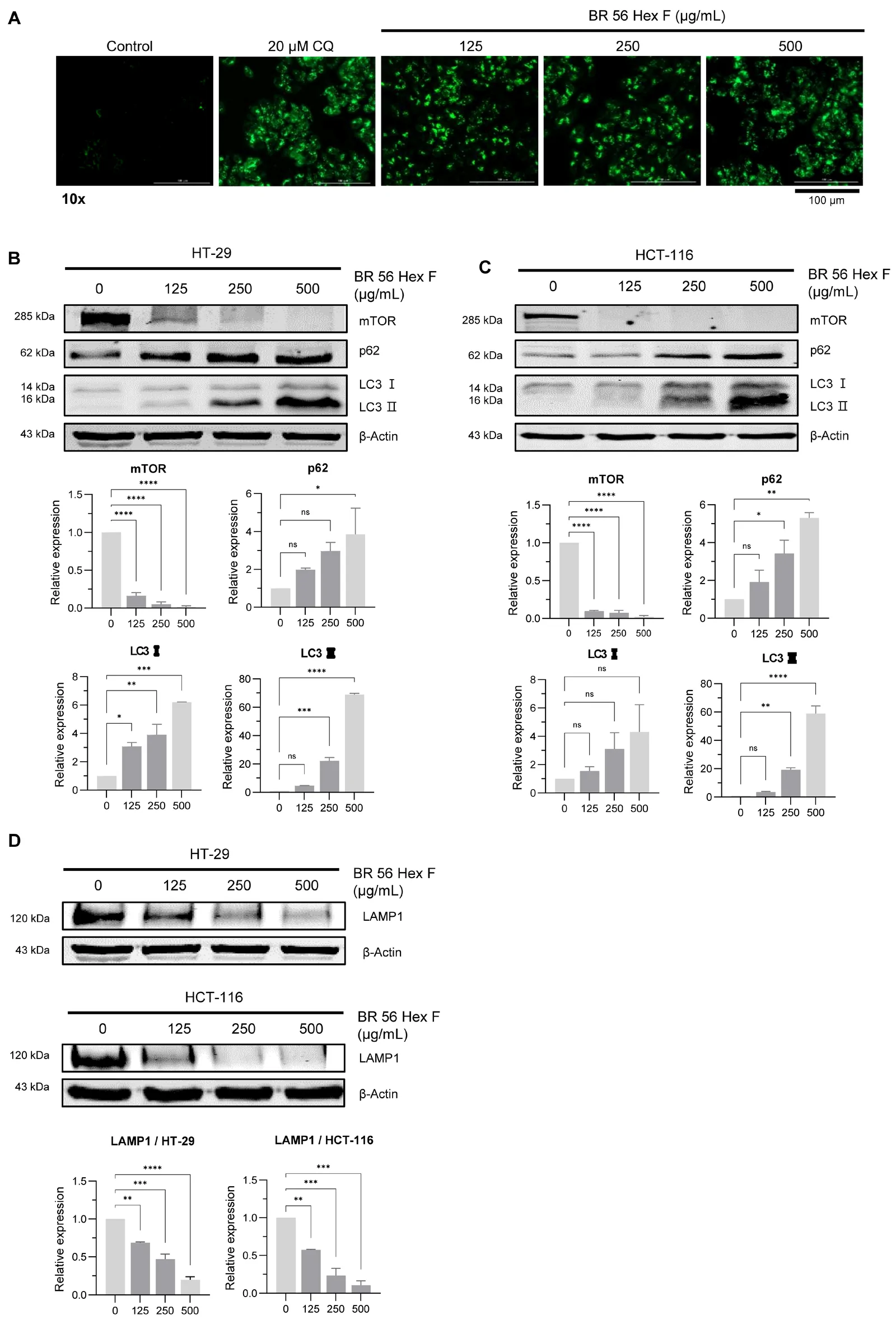
The hexane fraction of BR 56 Green *Carica papaya* L. impairs autophagy in HT-29 colon cancer cells. (**A**) Autophagosomes were visualized using Cyto-ID^®^ fluorescent dye in HT-29 cells treated with the BR 56 Hex F, chloroquine (20 μM), and analyzed by fluorescence microscopy. Magnification × 10; scale bar = 100 μm. (**B**) HT-29 cells were treated with BR 56 Hex F for 24 h, and the expression levels of the autophagy-related proteins mTOR, p62, LC3-I, and LC3-II were analyzed by Western blotting. Densitometric analysis was performed using Image Studio software. (**C**) HCT-116 cells were exposed to BR 56 Hex F for 24 h, after which mTOR, p62, LC3-I, and LC3-II protein expression was evaluated by Western blot analysis. Protein band intensities were subsequently quantified using Image Studio software. (**D**) The expression level of the lysosomal marker LAMP1 was detected by Western blotting following 24 h of treatment with BR 56 Hex F. Densitometric analysis was performed using Image Studio software. Data are presented as mean ± SD from three independent biological experiments (*n* = 3). Statistical significance was determined using one-way ANOVA followed by Tukey’s multiple comparisons test. * *p*  <  0.05, ** *p*  <  0.01, *** *p*  <  0.001, and **** *p*  <  0.0001 compared with the control.

Cyto-ID staining was performed to monitor autophagic activity after BR 56 Hex F treatment. It labels pre-autophagosomes and autophagosomes. Chloroquine (CQ) was used as the positive control, which is a known late-stage autophagy inhibitor. Compared with untreated cells, which exhibited weak green fluorescence, cells exposed to BR 56 Hex F showed a marked, concentration-dependent increase in green fluorescent puncta (Figure 3A). CQ-treated cells also showed increased green fluorescence, consistent with an impaired autophagy block. This supported the fact that BR 56 Hex F induces autophagosome accumulation by impairing autophagic flux. To further characterize the underlying mechanism, the expression of autophagy-associated proteins was examined by Western blotting. BR 56 Hex F treatment at 125, 250, and 500 μg/mL caused reduced mTOR expression (Figure 3B). mTOR is a nutrient-sensing kinase that regulates autophagy. These findings demonstrated that BR 56 Hex F inhibits mTOR and initiates autophagy. Based on these findings, we further checked the effect of BR 56 Hex F on LC3-II conversion. BR 56 Hex F treatment led to increased LC3-II protein levels in colon cancer cells in a dose-dependent manner (Figure 3B). p62 binds ubiquitinated proteins and facilitates their delivery to the autophagic machinery for degradation. Under normal autophagic conditions, p62 levels typically decline due to lysosomal degradation. Consequently, p62 accumulation may indicate impaired autophagic flux [15]. BR 56 Hex F increased the expression level of p62 protein, suggesting that BR 56 Hex F causes the late stage of the autophagy pathway (Figure 3B). To determine whether the autophagy-related effects of BR 56 Hex F extended beyond HT-29 cells, the same analyses were performed in HCT-116 cells. Immunoblot analysis revealed reduced mTOR protein levels along with increased LC3-II and p62 levels following treatment (Figure 3C). These findings are consistent with altered autophagy regulation and a possible impairment of autophagic degradation. Furthermore, LAMP1 expression was significantly reduced in BR 56 Hex F-treated cells (Figure 3D), suggesting a potential alteration in lysosomal function or abundance. Although the magnitude of changes in individual markers varied between the cell lines, the overall response patterns were consistent, supporting the reproducibility of BR 56 Hex F- induced alterations in autophagy-related processes across these two colorectal cancer cell lines. However, these differences in marker responses do not establish whether one cell line is more sensitive to BR 56 Hex F than the other.

### 3.4. The Hexane Fraction of BR 56 Carica papaya L. Impairs Autophagic Flux in HT-29 Colon Cancer Cells

**Figure 4:**
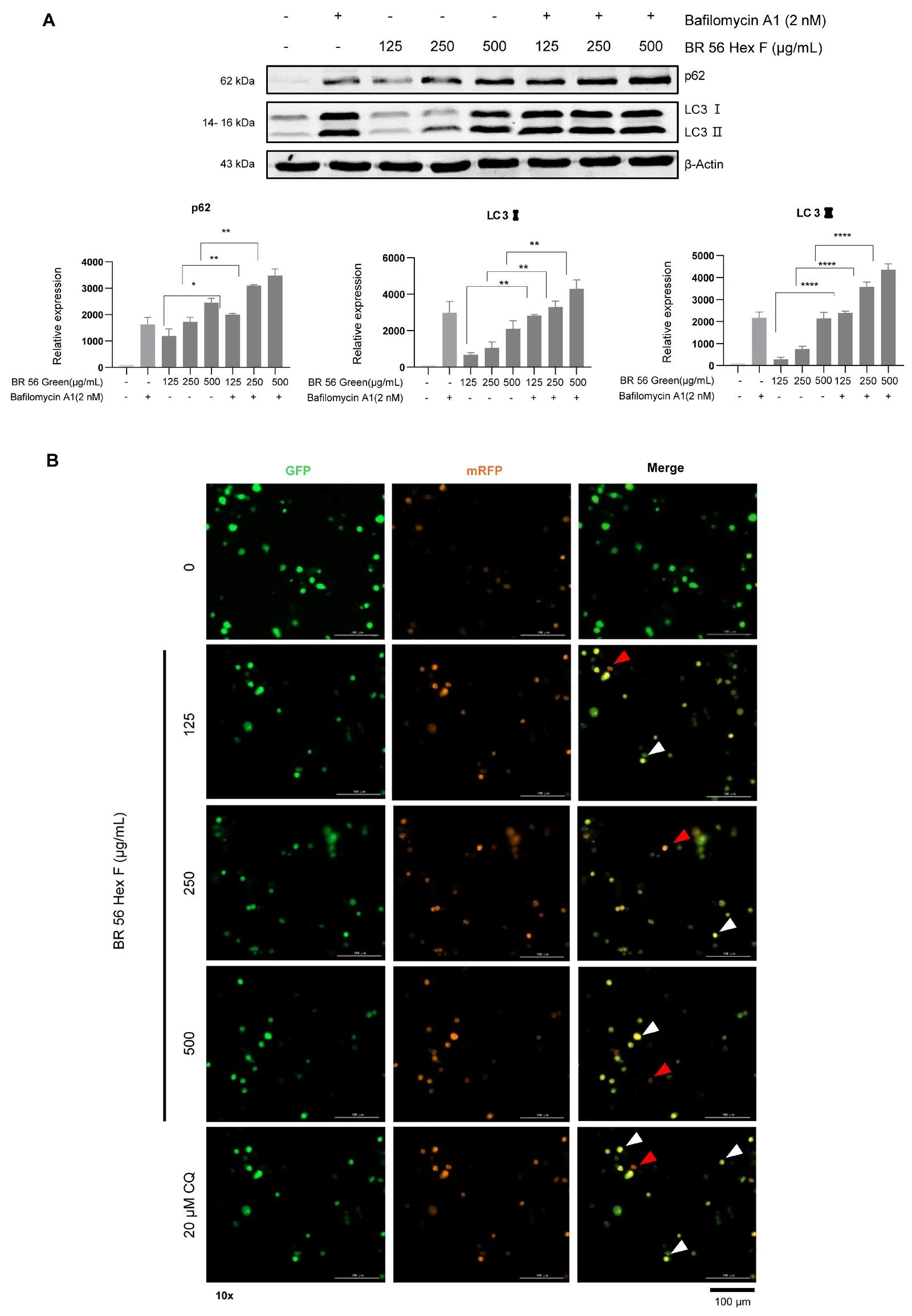
The hexane fraction of BR 56 Green *Carica papaya* L. impairs autophagic flux in HT-29 colon cancer cells. (**A**) HT-29 cells were exposed to BR 56 Hex F or bafilomycin A1 (BafA1) for 24 h. Western blotting was used to assess p62, LC3-I, and LC3-II protein levels, and band intensities were quantified using Image Studio software. (**B**) HT-29 cells transfected with the ptfLC3 reporter plasmid, followed by treatment with BR 56 Hex F for 24 h and examined by fluorescence microscopy. Images were captured at ×10 magnification; scale bar = 100 µm. White arrows demonstrate autophagosome accumulation/autophagic flux blockade (yellow puncta), and red arrows indicate autolysosomes (red-only puncta). Data are presented as mean ± SD from three independent biological experiments (*n* = 3). Statistical significance was determined using one-way ANOVA followed by Tukey’s multiple comparisons test. * *p*  <  0.05, ** *p*  <  0.01, and **** *p*  <  0.0001 compared with the control.

BafA1, a well-known pharmacological inhibitor of autophagy, was administered to further investigate whether BR 56 Hex F impairs autophagic flux. BafA1 inhibits vacuolar-type H^+^-ATPase (V-ATPase), thereby impairing lysosomal acidification and impairing lysosomal degradation, resulting in a late-stage autophagy block. In our study, BafA1 treatment alone increased LC3-II and p62 levels, consistent with the accumulation of autophagosomes due to blocked degradation. After 24 h of co-treatment with BR 56 Hex F and BafA1, we observed significantly greater LC3-II and p62 accumulation than with BR 56 Hex F treatment alone, supporting the involvement of impaired autophagic flux (Figure 4A). These results indicate that BR 56 Hex F disrupts autophagy, possibly during autophagosome–lysosome fusion or lysosomal function.

To further evaluate autophagic flux, cells were transiently transfected with the tandem fluorescent mRFP–EGFP–LC3 reporter (ptfLC3), which enables the differentiation of autophagosomes from acidic autolysosomal compartments. Because EGFP fluorescence is sensitive to acidic conditions, whereas mRFP fluorescence is relatively stable, autophagosomes exhibit both green and red fluorescence, appearing as yellow puncta in merged images. In contrast, red-only puncta indicate acidic autolysosomal compartments in which EGFP fluorescence is quenched [16]. As shown in Figure 4B, BR 56 Hex F-treated cells exhibited an increased abundance of yellow puncta accompanied by a relative decrease in red-only puncta. This pattern suggests the accumulation of non-acidified autophagic compartments and reduced progression toward acidic autolysosomal compartments. A similar distribution was observed in chloroquine (CQ)-treated cells, which served as a positive control for impaired lysosomal acidification and autophagic degradation. These findings suggest that BR 56 Hex F may impair late-stage autophagic processing, although further experiments are required to distinguish defects in autophagosome maturation, lysosomal fusion, and acidification.

### 3.5. BR 56 Carica papaya L. Hexane Fraction Suppresses the PI3K/AKT Signaling Pathway in Colon Cancer Cells

**Figure 5:**
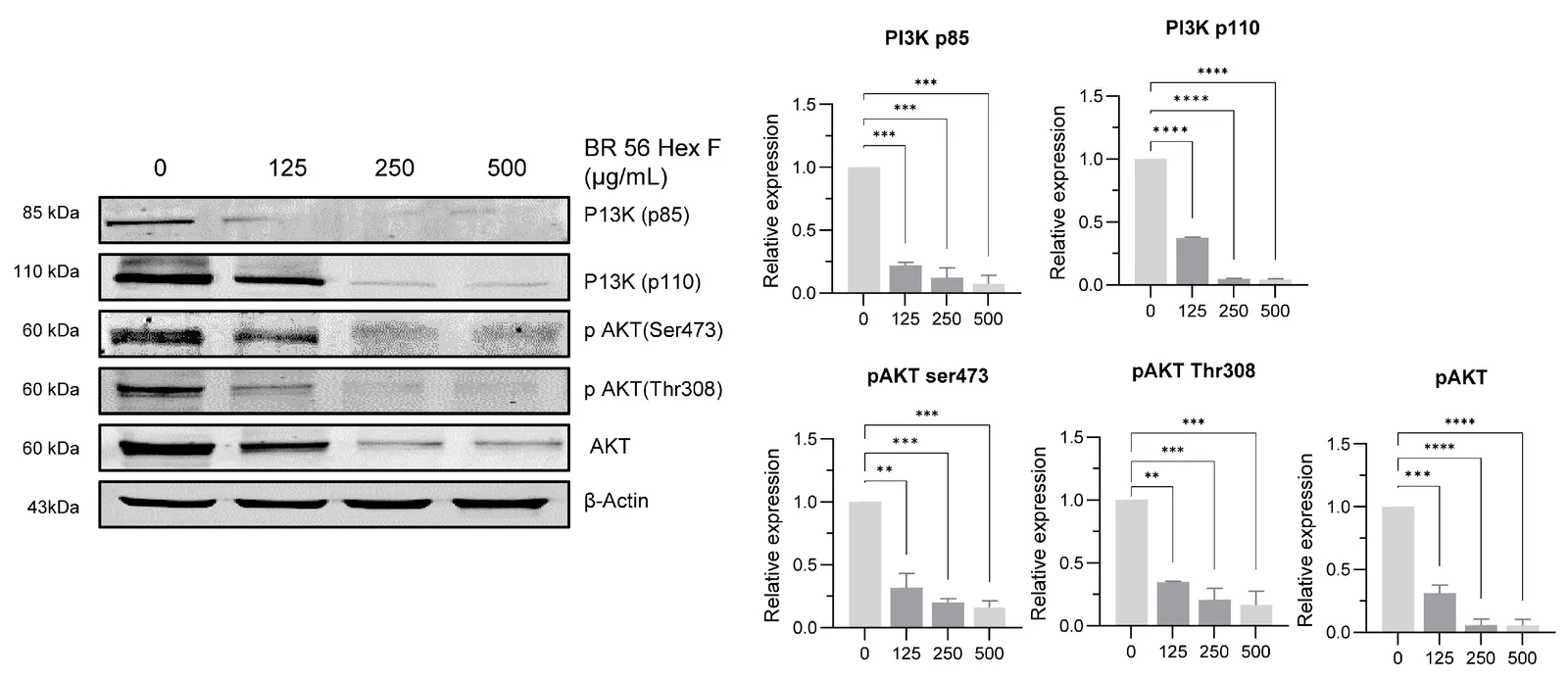
The hexane fraction of BR 56 Green *Carica papaya* L. suppresses the PI3K/AKT signaling pathway in colon cancer cells. HT-29 cells were treated with the BR 56 Hex F (125, 250, and 500 μg/mL) for 24 h. Protein expression levels of PI3K (p85), PI3K (p110), phosphorylated AKT (Ser473), phosphorylated AKT (Thr308), total AKT, and β-actin were analyzed by Western blotting. β-Actin was used as the loading control. Densitometric analysis was performed using Image Studio software. Data are presented as mean ± SD from three independent biological experiments (*n* = 3). Statistical significance was determined using one-way ANOVA followed by Tukey’s multiple comparisons test. ** *p*  <  0.01, *** *p*  <  0.001, and **** *p*  <  0.0001 compared with the control.

We examined the effects of BR 56 Hex F on the PI3K/AKT signaling pathway in HT-29 colon cancer cells to investigate the molecular mechanisms underlying its anticancer activity. Treatment with BR 56 Hex F (125, 250, and 500 μg/mL) markedly decreased the expression of PI3K regulatory and catalytic subunits, including PI3K p85 and PI3K p110, in a concentration-dependent manner. Furthermore, BR 56 Hex F significantly decreased the phosphorylation levels of AKT at the Ser473 and Thr308 residues, along with a reduction in total AKT expression at higher concentrations. Quantitative densitometric analysis confirmed the progressive suppression of PI3K/AKT pathway activation following BR 56 Hex F treatment compared to that in untreated control cells (Figure 5). Given that PI3K/AKT signaling plays a key role in modulating CRC cell proliferation, survival, metabolism, and resistance to apoptosis, the observed inhibition of PI3K/AKT phosphorylation suggests that BR 56 Hex F exerts its anticancer effects, at least in part, through the suppression of this oncogenic pathway. These findings indicate that BR 56 Hex F may interfere with PI3K/AKT-mediated survival signaling, contributing to the inhibition of HT-29 CRC cell growth.

### 3.6. Effect of BR 56 Carica papaya L. Oral Administration on the AOM/DSS Treated ICR Mice

**Figure 6:**
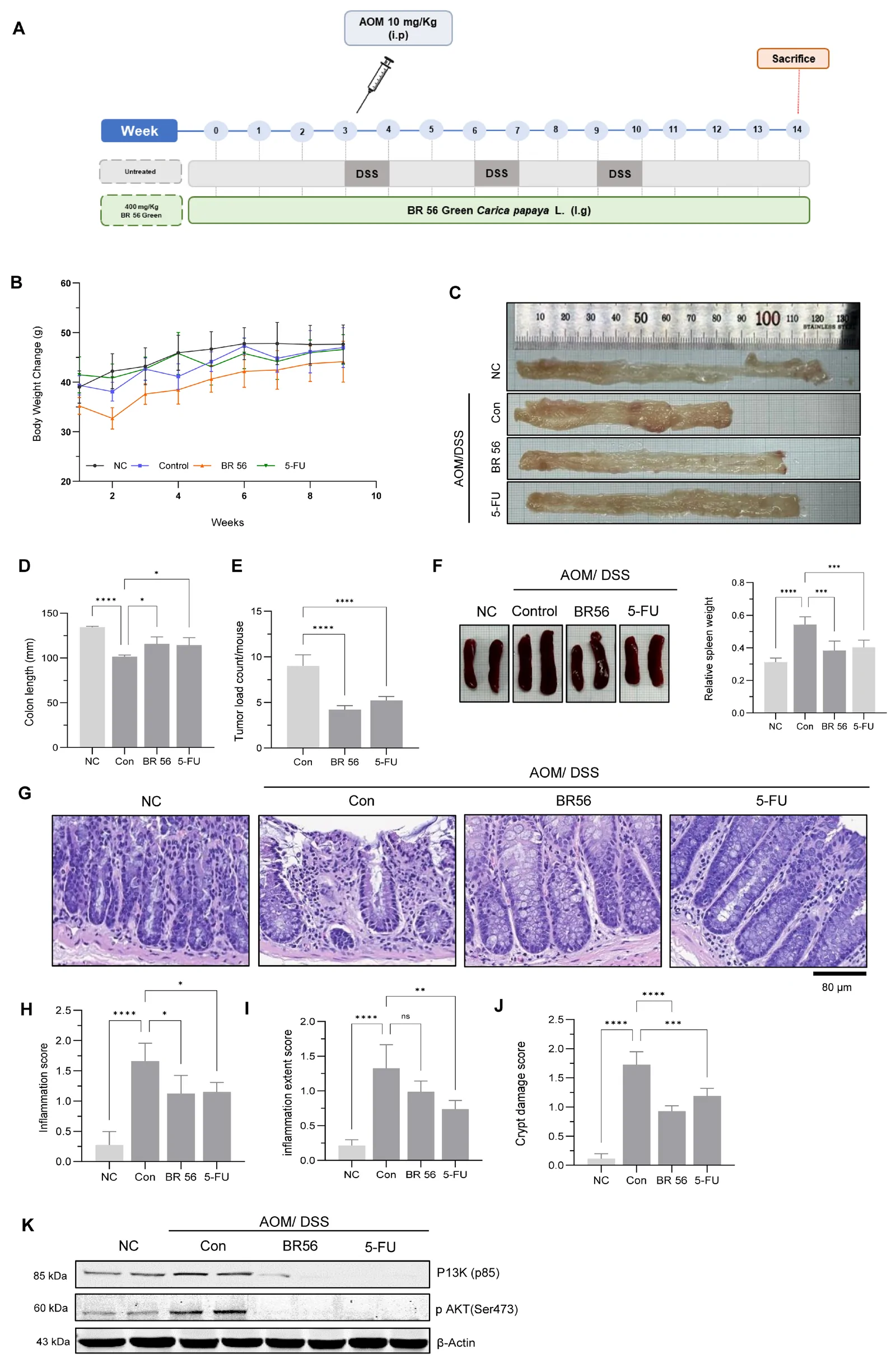
BR 56 Green *Carica papaya* L. extracts attenuate AOM/DSS-induced colitis and systemic inflammation. (**A**) Experimental design and timeline for AOM/DSS-induced colorectal carcinogenesis in ICR mice, including oral administration schedules for BR 56 Green *C. papaya* L. extract (400 mg/kg, i.g.). (**B**) Weekly body weight changes in mice across experimental groups during the treatment period. (**C**) Representative gross images of colons harvested from the Negative Control, AOM/DSS Control, BR 56 Green *C. papaya* L. extract, and 5-fluorouracil groups. (**D**) Quantification of colon length (mm) across treatment groups. (**E**) Total tumor count per mouse colon across experimental groups. (**F**) Representative macroscopic images of spleens and quantification of relative spleen weight. (**G**) Representative hematoxylin and eosin (H&E)-stained colon sections showing the effects of BR 56 Green *C. papaya* L. extract on AOM/DSS-induced histopathological alterations. (**H**–**J**) Histopathological scores for inflammation severity (**H**), inflammation extent (**I**), and crypt damage (**J**) in colon tissues. (**K**) Western blot analysis of PI3K (p85) and phosphorylated AKT (Ser473) expression in colon tissues from the indicated experimental groups. β-Actin was used as the loading control. Statistical significance was assessed by one-way analysis of variance (ANOVA) followed by Tukey’s multiple-comparisons test. * *p*  <  0.05, ** *p*  <  0.01, *** *p*  <  0.001, and **** *p*  <  0.0001, compared with the AOM/DSS control group; ns, not significant.

Mice were subjected to an AOM/DSS-induced colorectal carcinogenesis protocol and treated orally with BR 56 papaya extract throughout the experimental period to evaluate the chemopreventive effects of papaya extract against CRC (Figure 6A). Body weight was monitored weekly to assess the general health status of the animals during treatment (Figure 6B). Mice in the AOM/DSS control (Con) group exhibited an initial decrease in body weight following DSS administration compared with the negative control group (NC). However, body weight gradually recovered during the later stages of the experiment. Throughout the study, mice receiving BR 56 extract orally maintained body weights comparable to those of the AOM/DSS control animals, with no obvious treatment-associated adverse effects. The 5-fluorouracil (5-FU) group also showed no statistically significant reduction in body weight, suggesting that both treatments were well tolerated under the experimental conditions.

Colon shortening is a common feature of colitis-associated colon cancer in AOM/DSS-induced CRC models [17]. Compared with the NC group (134.5 ± 1.0 mm), the colon length was markedly decreased in the control group (101.5 ± 1.9 mm). Conversely, treatment with the BR 56 *C. papaya* L. extract significantly attenuated AOM/DSS-induced colon shortening, increasing the mean colon length to 115.8 ± 7.8 mm compared with 101.5 ± 1.9 mm in the AOM/DSS control group (*p* < 0.05). The 5-FU-treated group showed a comparable mean colon length of 114.3 ± 8.5 mm, significantly greater than the AOM/DSS control group (*p* < 0.05) (Figure 6D). In addition, the number of tumors was significantly higher in the AOM/DSS control group than in the NC group, whereas oral administration of the BR 56 extract significantly reduced the number of tumors compared to the AOM/DSS control group (Figure 6E). A similar reduction in tumor number was observed in the 5-FU-treated group. Quantitative analysis revealed that the CRC control group developed an average of 9.00 ± 1.22 tumors per mouse, whereas the BR 56 and 5-FU treatment groups developed 4.20 ± 0.45 and 5.20 ± 0.45 tumors per mouse, respectively. These findings indicate that the BR 56 extract suppressed AOM/DSS-induced tumor development in the colon.

Splenomegaly is a hallmark of systemic inflammation associated with AOM/DSS-induced colorectal carcinogenesis. As shown in Figure 6F, the relative spleen weight was significantly higher in the AOM/DSS control group than that in the negative control group, indicating enhanced systemic inflammatory responses. Treatment with BR 56 papaya extract significantly reduced the relative spleen weight compared to the AOM/DSS control group. A similar reduction was observed in the 5-FU-treated group. These findings suggest that the BR 56 papaya extract attenuated AOM/DSS-induced systemic inflammation.

Histological analysis of the colon tissues using hematoxylin and eosin (H&E) staining further demonstrated the protective effect of BR 56 treatment (Figure 6G). The NC group exhibited an intact mucosal architecture and well-organized colonic crypts, whereas the AOM/DSS control group displayed substantial inflammatory cell infiltration, mucosal damage, and crypt disruption. BR 56 treatment markedly attenuated these pathological alterations, resulting in improved preservation of colonic architecture. Similar histological improvements were observed in the 5-FU-treated groups. Consistent with these morphological observations, the inflammation score increased from 0.28 ± 0.22 in the Negative control group to 1.66 ± 0.29 following AOM/DSS administration. BR 56 treatment reduced the inflammation score to 1.13 ± 0.30, whereas the 5-FU-treated group showed a comparable score of 1.15 ± 0.15 (Figure 6H). The inflammation extent and crypt damage scores were also significantly increased in the AOM/DSS control group, whereas both parameters were significantly reduced following BR 56 treatment (Figure 6I,J).

The protein expression levels of PI3K (p85) and phosphorylated AKT (p-AKT; Ser473) in colonic tissues were investigated to determine whether the BR 56 *C. papaya* L. fruit extract attenuated colitis-associated colorectal cancer (CAC) by modulating the PI3K/AKT signaling pathway (Figure 6K). As shown in Figure 6K, AOM/DSS treatment increased the expression of PI3K (p85) and p-AKT (Ser473) compared to that in the negative control group. Treatment with BR 56 *C. papaya* L. extract reduced PI3K (p85) and p-AKT (Ser473) expression compared to the AOM/DSS control group. Similarly, the positive control treatment with 5-fluorouracil also reduced the activation of the PI3K/AKT signaling pathway.

## 4. Discussion

CRC is a global health concern that warrants the development of effective therapeutics that can efficiently suppress tumor growth while minimizing treatment-related toxicity. Natural compounds obtained from medicinal and edible plants have gained attention as promising sources of anticancer agents, because they contain structurally diverse bioactive compounds with various bioactivities. In this study, we investigated the anticancer potential and underlying molecular mechanisms of the hexane fraction from BR 56 *C. papaya* L. fruit grown in Jeju, Republic of Korea, which shows potent anticancer activity against CRC in vitro and in vivo. Our findings demonstrated that BR 56 Hex F drives CRC cell apoptosis by executing a dual molecular mechanism: suppressing the oncogenic PI3K/AKT axis while paradoxically inducing a late-stage blockade of autophagic flux.

Previous research evaluating the anticancer potential of *C. papaya* L. has established its broad chemopreventive and antineoplastic capabilities against various human malignancies, including breast, prostate, liver, and colorectal cancers. In CRC models, crude aqueous and alcoholic seed extracts of *C. papaya* L. cultivated in Egypt have been shown to trigger G2/M phase cell cycle arrest and intrinsic apoptosis via p53 activation, cytochrome release, and cleaved caspase 3 activation, with downregulation of anti-apoptotic Bcl-2 family members [18]. Similarly, leaf and pulp extracts of *C. papaya* L. cultivated in India reportedly suppress colon cancer cell proliferation and migration by modulating Wnt/β-catenin signaling and apoptotic pathways. In another study, methanol extract of ripened *C. papaya* L. cultivated in India showed a growth-inhibitory effect on HT-29 cells, accompanied by increased ROS levels, reduced cell migration, and decreased clonogenic potential. However, the existing literature on *C. papaya* L. in cancer research has mainly focused on crude polar extracts derived from tropical varieties [19].

Phytochemical studies of *C. papaya* L. cultivated in different geographical regions have also demonstrated variations in their phytochemical composition among plant parts and cultivars. A study on Indonesian papaya reported differences in total phenolic and flavonoid contents among pulp, peel, and seed extracts prepared using solvents of different polarities [20]. Furthermore, a study of red and yellow papaya varieties cultivated in Thailand identified diverse phenolic compounds including caffeic acid, gallic acid, ferulic acid, catechin, gallocatechin, quercetin derivatives, and kaempferol derivatives. The distribution of these phenolic compounds varied among the peel, pulp, and seed extracts prepared using solvents of different polarities [21]. Another study on *C. papaya* L. cultivated in India identified 5-hydroxy feruloyl quinic acid, syringic acid hexoside, acetyl p-coumaryl quinic acid, quercetin-3-O-rhamnoside, 5-hydroxy caffeic quinic acid, peonidin-3-O-glucoside, cyanidin-3-O-glucoside, sinapic acid-O-hexoside, and methyl feruloyl glycoside in papaya aqueous extracts using LC–MS/MS [22]. In contrast to previous studies on *C. papaya* L., which mainly investigated crude polar extracts from tropical cultivars, our study focused on the nonpolar hexane fraction (BR 56 Hex F) prepared from immature BR 56 fruit grown in Korea. Consistent with previous reports of papaya extract-induced ROS production and apoptosis, our study also demonstrated increased ROS production and apoptosis induction. However, the involvement of *C. papaya* L. extracts in autophagy regulation and oncogenic signaling pathways like PI3K/AKT remains poorly explored. These comparisons provide context for our findings but do not establish greater potency or cultivar-specific effects, because the studies differ in plant materials, extraction methods, and experimental conditions.

The PI3K/AKT pathway is activated in approximately 60% of cancer cases and serves as the primary regulator of cell proliferation, metabolic adaptation, and chemoresistance [23]. Pharmacological suppression of this signaling cascade often triggers adaptive survival mechanisms such as autophagy. Typically, the suppression of upstream PI3K/AKT/mTOR signaling removes the inhibitory restriction of ULK1, initiating early autophagic nucleation to recycle nutrients and protect organelle integrity [24]. Consistent with this scenario, we found that BR 56 Hex F administration significantly downregulated the catalytic (p110) and regulatory (p85) subunits of PI3K, decreased the downstream phosphorylation of AKT (Ser473/Thr308), and inhibited mTOR activity. These findings indicate that BR 56 Hex F disrupts the PI3K/AKT/mTOR survival axis in CRC cells. Despite suppression of PI3K/AKT/mTOR signaling, BR 56 Hex F did not exhibit effective autophagic degradation. Rather, several observations suggest that autophagic flux is impaired at later stages.

Evidence from previous research suggests that pharmacological inhibition of PI3K-dependent survival pathways may contribute to apoptosis in colorectal cancer cells [25,26]. Mechanistically, AKT promotes cell survival by suppressing intrinsic mitochondrial apoptotic signaling and activating the pro-apoptotic proteins Bax and Bak. Inhibition of PI3K/AKT signaling may therefore weaken these pro-survival mechanisms and facilitate mitochondrial outer membrane permeabilization, cytochrome c release, and subsequent caspase activation [27]. These mechanisms provide a biological rationale for a possible link between PI3K/AKT suppression and apoptosis in our study. However, we did not directly assess Bax/Bak activation or cytochrome c release. Consistent with this interpretation, BR 56 Hex F treatment increased the levels of cleaved caspase-9 and cleaved caspase-7, suggesting the involvement of intrinsic apoptotic signaling. Collectively, these findings suggest that suppression of PI3K/AKT/mTOR-mediated survival signaling may contribute to BR 56 Hex F-induced apoptosis, although the concurrent changes in signaling and apoptosis markers do not establish causality. Further mechanistic studies are required to determine whether PI3K/AKT suppression is necessary for the apoptosis response.

LC3-II is associated with autophagosomal membranes and is commonly used to assess autophagosome abundance. However, increased LC3-II levels alone cannot distinguish enhanced autophagosome formation from impaired autophagosome degradation. Therefore, we examined p62/SQSTM1 as an additional marker of autophagic degradation. p62/SQSTM1 functions as a selective autophagy cargo receptor that binds ubiquitinated proteins and facilitates their delivery to autophagosomes for subsequent lysosomal degradation. Under normal autophagic conditions, p62 is degraded through the autophagy–lysosome pathway; thus, its accumulation may indicate impaired autophagic degradation [15]. In the present study, BR 56 Hex F treatment increased both LC3-II and p62/SQSTM1 levels in a concentration-dependent manner, suggesting that autophagosome accumulation was accompanied by reduced autophagic degradation. These findings led us to investigate whether lysosomal dysfunction contributed to the observed late-stage impairment of autophagic flux. To further investigate the potential involvement of lysosomes in this process, we examined lysosome-associated membrane protein 1 (LAMP1), a major lysosomal membrane protein associated with lysosomal integrity and function. BR 56 Hex F treatment reduced LAMP1 protein expression, suggesting altered lysosomal abundance or homeostasis, although this measurement alone does not establish lysosomal dysfunction. To further assess autophagic flux, we performed tandem fluorescent mRFP–EGFP–LC3 reporter analysis. BR 56 Hex F-treated cells exhibited an increased abundance of yellow puncta accompanied by a relative decrease in red-only puncta, indicating the accumulation of non-acidified autophagic compartments and reduced progression toward acidic autolysosomal compartments. A similar pattern was observed in chloroquine-treated cells, supporting the possibility that BR 56 Hex F impairs late-stage autophagic processing.

Taken together, these findings suggest that BR 56 Hex F suppresses PI3K/AKT/mTOR signaling while simultaneously disrupting the downstream completion of autophagy, resulting in a late-stage autophagy block. Similar findings have been reported for 4-acetylantroquinonol B in chemoresistant ovarian cancer cells, where inhibition of the PI3K/AKT/mTOR/p70S6K pathway was accompanied by the suppression of autophagic flux and enhanced cancer cell death [14]. The antimicrobial peptide WK-13-3D also reportedly inhibits AKT/mTOR signaling while causing LC3 II accumulation and p62 stabilization, and impairing the progression of autophagosomes to autolysosomes [13]. These findings further indicate that inhibition of the AKT/mTOR signaling pathway can occur alongside the disruption of autophagic flux in cancer cells.

Similar changes in autophagy-associated proteins were also observed in HCT-116 cells treated with BR 56 Hex F, including reduced mTOR expression and increased levels of LC3-II and p62. The consistency of these changes across both colorectal cancer cell models supports the reproducibility of the observed response and indicates that BR 56 Hex F may interfere with autophagic degradation rather than producing a cell-line-specific effect.

BR 56 Hex F regulates PI3K/AKT inhibition, impairs autophagy, and promotes mitochondrial apoptosis. Furthermore, BR 56 Hex F promotes ROS production and accumulation of mitochondrial ROS. The elevated ROS levels in BR 56 Hex F-treated cells may originate from several sources, including mitochondrial dysfunction and proteotoxic stress associated with the accumulation of undegraded protein aggregates following autophagy inhibition. These processes may collectively exacerbate intracellular oxidative stress.

Our findings further showed increased activation of cleaved caspase-9 and cleaved caspase-7 following BR 56 Hex F treatment, whereas cleaved caspase-3 was not detected despite the presence of total caspase-3. Cleaved caspase-9 is the key initiator of the intrinsic mitochondrial apoptotic pathway and subsequently activates executioner caspases, including cleaved caspase-3 and cleaved caspase-7. The activation of cleaved caspase-7 observed in our study may be associated with mitochondrial apoptotic signaling induced by BR 56 Hex F. However, the absence of detectable cleaved caspase-3 under the conditions examined does not exclude its involvement. Genetic and molecular background can influence apoptosis responses in colorectal cancer cells. Nevertheless, the absence of detectable cleaved caspase-3 under the conditions examined does not necessarily exclude its involvement in the apoptotic response. HT-29 cells have been reported to exhibit deficient caspase-3 expression or activity, which may influence their apoptotic response and favor caspase-7-mediated execution of cell death. Therefore, the observed caspase cleavage pattern may partly reflect the intrinsic molecular characteristics of HT-29 cells rather than a BR 56 Hex F-specific alternative apoptotic mechanism. However, this possibility was not directly investigated in the present study [28,29].

We then evaluated the in vivo chemopreventive efficacy of BR 56 *C. papaya* L. extract in an AOM/DSS-induced colitis-associated CRC mouse model. The in vivo and in vitro experiments used different preparations, namely BR 56 Hex F and BR 56 *C. papaya* L. extract, respectively. Therefore, the animal findings do not directly establish the in vivo efficacy or mechanism of the hexane fraction. Oral administration of the BR 56 extract significantly attenuated colon shortening and reduced relative spleen weights (splenomegaly) to levels comparable to those of the standard chemotherapeutic agent 5- FU, without inducing systemic toxicity or adverse effects on body weight. Histological analysis provided additional evidence of tissue protection, with BR 56 treatment reducing inflammatory cell infiltration, mucosal damage, crypt disruption, and the histological score. These effects were consistent with those observed in the 5-FU-treated group.

The PI3K/AKT signaling pathway is frequently dysregulated in colorectal cancer and contributes to tumor-cell survival, proliferation, and resistance to apoptosis [30]. Consequently, targeting this pathway has been investigated as a potential strategy for colorectal cancer prevention and treatment. Furthermore, BR 56 treatment reduced the expression of PI3K p85 and phosphorylated AKT (Ser473) in colonic tissues, supporting the association between reduced PI3K/AKT signaling and the observed in vivo effect. Previous studies using AOM/DSS induced CRC models have similarly reported that suppression of PI3K/AKT signaling is associated with reduced tumor development and modulation of inflammation-related pathways. For example, Theabrownin (TB) treatment was reported to suppress PI3K/AKT/mTOR signaling and reduce colorectal tumor burden [31]. Furthermore, in another study, wogonoside attenuated inflammation-associated colorectal carcinogenesis through modulation of PI3K/AKT-mediated NF-κB activation [32]. These findings are consistent with our in vivo observations and suggest that modulation of the PI3K/AKT pathway may contribute to the chemopreventive effects of BR 56 *C. papaya* L. extracts in colitis-associated CRC. As BR 56 administration began on the day of AOM injection, these results primarily support its preventive potential during colorectal carcinogenesis rather than demonstrate efficacy against established tumors.

To our knowledge, this study provides the first evidence that the hexane fraction obtained from immature BR 56 *Carica papaya* L. cultivated in Jeju exhibits anticancer activity against colorectal cancer. In vitro, this activity is mediated through PI3K/AKT/mTOR suppression, impaired late-stage autophagic flux, and induction of mitochondrial apoptosis. In the animal model, administration of BR 56 *C. papaya* L. extract attenuated AOM/DSS-induced inflammation and tissue injury, accompanied by reduced levels of PI3K (p85) and phosphorylated AKT in colonic tissue. However, these findings do not establish impaired autophagic flux or mitochondrial apoptosis in vivo. These findings provide novel mechanistic insights into the anticancer potential of locally cultivated *C. papaya* L. and support further investigation of BR 56 Hex F as a potential natural chemopreventive candidate.

Several limitations of the present study should be acknowledged. First, BR 56 Hex F is a complex mixture of various bioactive compounds. Therefore, further bioactivity-guided fractionation and structural characterization are needed to identify the active constituents and clarify their individual or combined contributions to the observed anticancer activity. Second, we did not investigate the potential synergistic effects of BR 56 Hex F with clinically used chemotherapeutic agents, such as 5-fluorouracil or oxaliplatin. Further studies are needed to determine whether BR 56 Hex F can enhance the efficacy of these agents in CRC. Finally, although the in vivo study demonstrated the anticancer effects of BR 56 extract in the AOM/DSS-induced CRC model, comprehensive toxicity and pharmacokinetic assessments were not performed. Further in vivo studies using BR 56 Hex F are needed to evaluate its safety, pharmacokinetic properties, and therapeutic potential.

## 5. Conclusions

Our study provides a comprehensive molecular and in vivo framework demonstrating the potent antitumor activity of the hexane fraction of Jeju-cultivated *C. papaya* L. (BR 56) against CRC. By simultaneously silencing the oncogenic PI3K/AKT/mTOR survival axis and crippling late-stage autophagic flux, BR 56 Hex F converts an adaptive cellular recycling process into an unresolvable state of organelle stress, proteotoxicity, and ROS accumulation. This organelle and oxidative crisis drive CRC cells toward a caspase-9/caspase-7-mediated intrinsic apoptotic pathway. Furthermore, the BR 56 extracts effectively suppressed colitis-associated colorectal carcinogenesis in vivo and attenuated systemic inflammation without any observable toxicity. Collectively, these findings highlight the therapeutic potential of the lipophilic fractions from domestically cultivated *C. papaya* L.as novel natural chemopreventive candidates or adjuvant therapeutic agents for colorectal malignancies.

## Data Availability

The original contributions presented in this study are included in the article/Supplementary Materials.

## Supplementary Materials

The following supporting information can be downloaded at: https://www.mdpi.com/article/10.3390/nu18213557/s1, Figure S1: Effects of selected solvent fractions of BR 56 Green *Carica papaya* L. fruit on the viability of HT-29 cells; Figure S2: Effects of selected solvent fractions of BR 56 Yellow *Carica papaya* L. fruit on the viability of HT-29 cells.

## Abbreviations

The following abbreviations are used in this manuscript:

CRC	Colorectal cancer
PI3K	Phosphoinositide 3-kinase
AKT	Protein kinase B (AKT)
mTOR	Mechanistic target of rapamycin
ROS	Reactive oxygen species
HT-29	Cell-line designation; use this format consistently
HCT-116	Cell-line designation; use this format consistently
p62	Sequestosome 1 (SQSTM1/p62); define at first use
LAMP1	Lysosome-associated membrane protein 1
BafA1	Bafilomycin A1
5-FU	5-Fluorouracil
mRFP-EGFP-LC3	Monomeric red fluorescent protein–enhanced green fluorescent protein–LC3 reporter
AOM	Azoxymethane
DSS	Dextran sulfate sodium
i.p.	Intraperitoneal
i.g.	Intragastric
ANOVA	Analysis of variance
SD	Standard deviation
