# Supplementary material for: Carica papaya L. Hexane Fraction Induces Apoptotic Cell Death in Colorectal Cancer Cells via PI3K/AKT Suppression and Impaired Late-Stage Autophagic Flux
Source: Nutrients. 2026 Oct 28;18(21):3557. doi: 10.3390/nu18213557 (PMC13764734; doi:10.3390/nu18213557)
Supplement: Supplementary file 1 [file nutrients-18-03557-s001.zip › nutrients-4627426-supplementary.pdf]

Carica papaya L. Hexane Fraction Induces Apoptotic Cell Death in Colorectal Cancer Cells via PI3K/AKT Suppression and Impaired Late-Stage Autophagic Flux.

Supplementary data

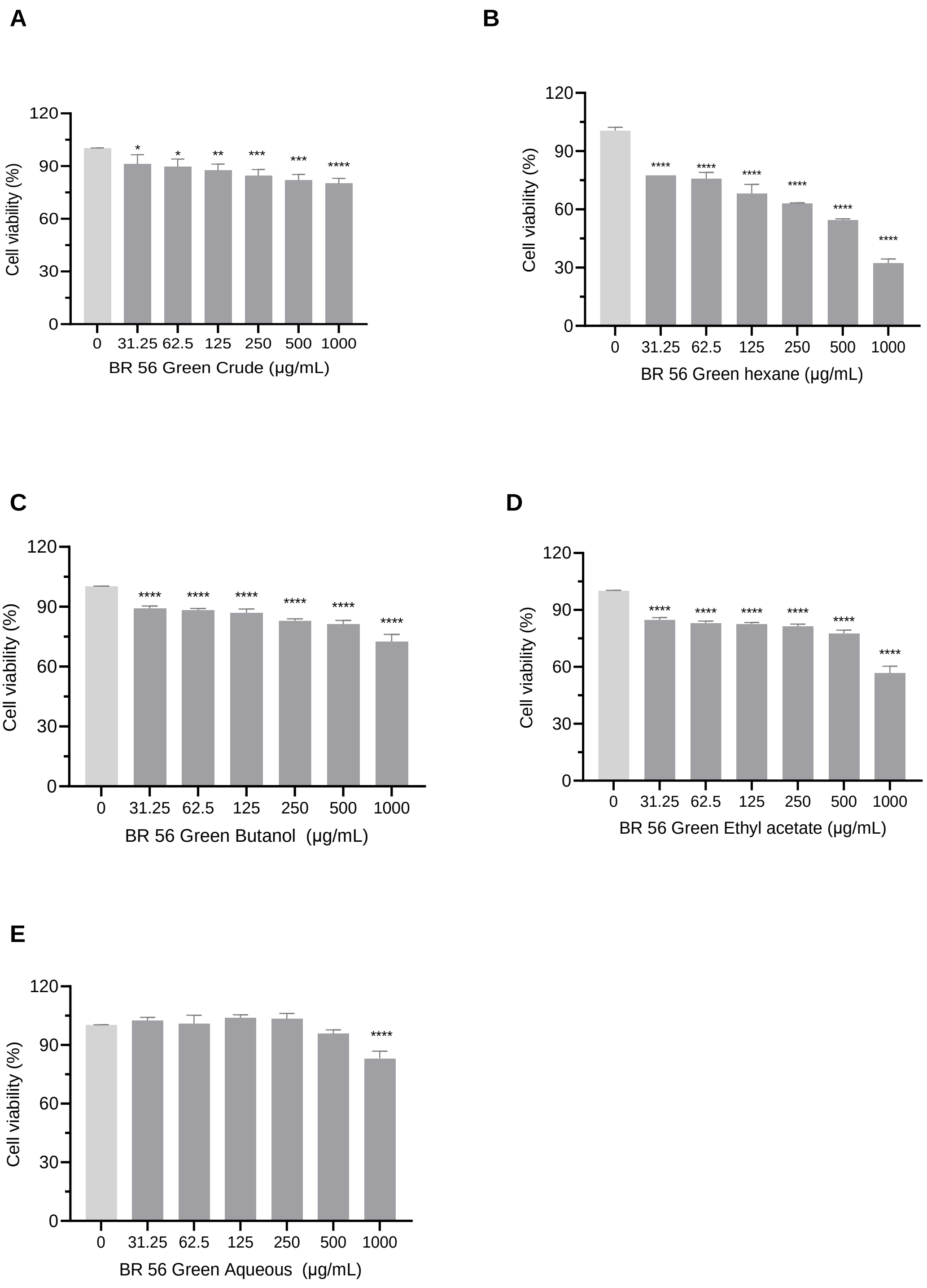

Figure S1. Effects of selected solvent fractions of BR 56 Green *Carica papaya* L. fruit on the viability of HT-29 cells. (A–F) Effects of the selected solvent fractions on the viability of HT-29 cells after 24 h of treatment, as determined by the WST assay. Statistical significance was determined using one-way ANOVA followed by Tukey's multiple comparisons test. *$P < 0.05$, **$P < 0.01$, ***$P < 0.001$, and ****$P < 0.0001$ compared with the control.

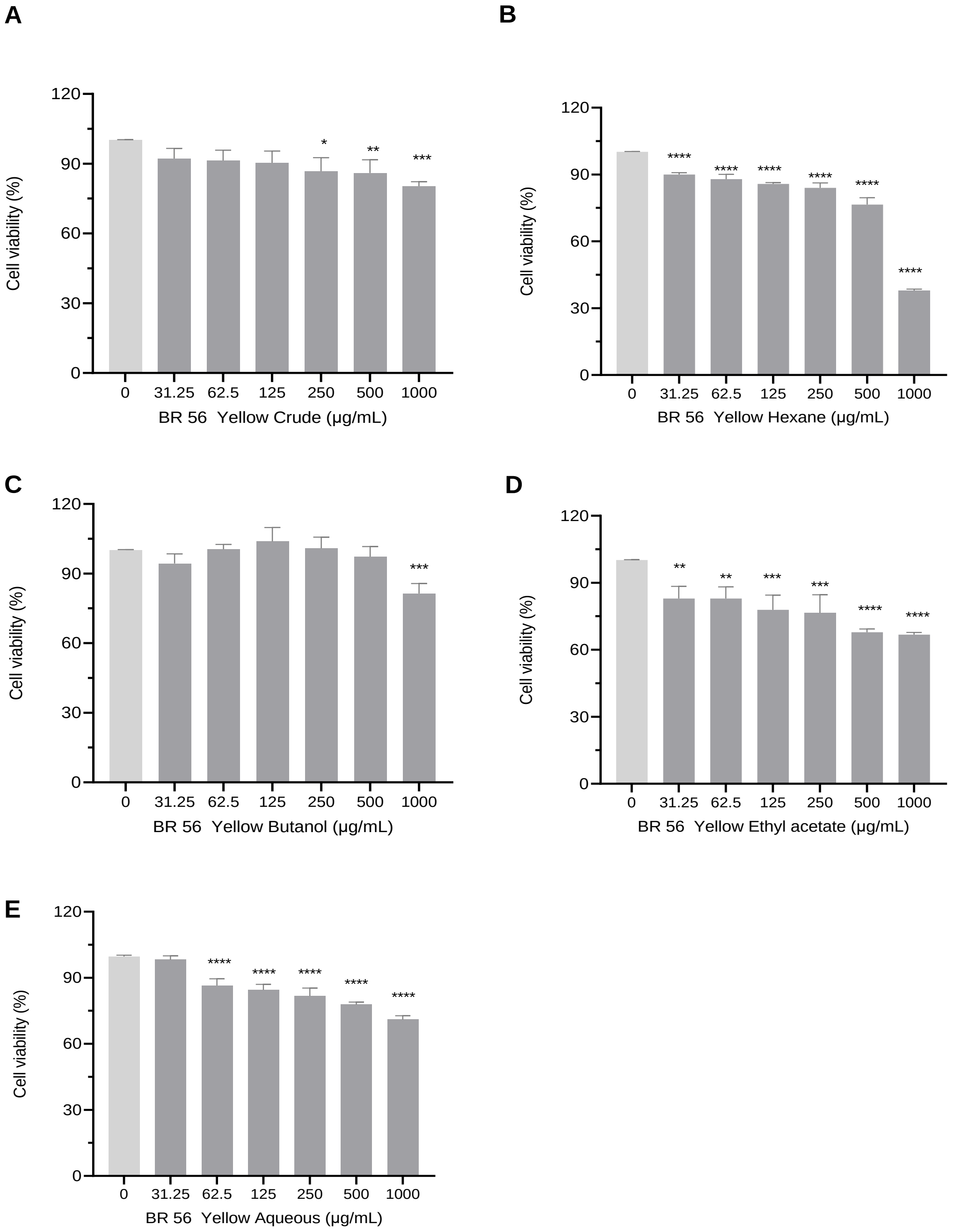

Figure S2. Effects of selected solvent fractions of BR 56 Yellow *Carica papaya* L. fruit on the viability of HT-29 cells. (A–F) Effects of the selected solvent fractions on the viability of HT-29 cells after 24 h of treatment, as determined by the WST assay. Statistical significance was determined using one-way ANOVA followed by Tukey's multiple comparisons test. *$P < 0.05$, **$P < 0.01$, ***$P < 0.001$, and ****$P < 0.0001$ compared with the control.
